# Exogenously Applied Sodium Nitroprusside Mitigates Lead Toxicity in Rice by Regulating Antioxidants and Metal Stress-Related Transcripts

**DOI:** 10.3390/ijms23179729

**Published:** 2022-08-27

**Authors:** Waqas Rahim, Murtaza Khan, Tiba Nazar Ibrahim Al Azzawi, Anjali Pande, Nusrat Jahan Methela, Sajid Ali, Muhammad Imran, Da-Sol Lee, Geun-Mo Lee, Bong-Gyu Mun, Yong-Sun Moon, In-Jung Lee, Byung-Wook Yun

**Affiliations:** 1 Department of Applied Biosciences, Kyungpook National University, Daegu 41566, Korea; 2 Department of Horticulture and Life Science, Yeungnam University, Gyeongsan 38541, Korea

**Keywords:** nitric oxide, antioxidants, metal-stress related transcripts, rice, Pb-stress

## Abstract

Sustainable agriculture is increasingly being put in danger by environmental contamination with dangerous heavy metals (HMs), especially lead (Pb). Plants have developed a sophisticated mechanism for nitric oxide (NO) production and signaling to regulate hazardous effects of abiotic factors, including HMs. In the current study, we investigated the role of exogenously applied sodium nitroprusside (SNP, a nitric oxide (NO) donor) in ameliorating the toxic effects of lead (Pb) on rice. For this purpose, plants were subjected to 1.2 mM Pb alone and in combination with 100 µM SNP. We found that under 1.2 mM Pb stress conditions, the accumulation of oxidative stress markers, including hydrogen peroxide (H_2_O_2_) (37%), superoxide anion (O_2_^−^) (28%), malondialdehyde (MDA) (33%), and electrolyte leakage (EL) (34%), was significantly reduced via the application of 100 µM SNP. On the other hand, under the said stress of Pb, the activity of the reactive oxygen species (ROS) scavengers such as polyphenol oxidase (PPO) (60%), peroxidase (POD) (28%), catalase (CAT) (26%), superoxide dismutase (SOD) (42%), and ascorbate peroxidase (APX) (58%) was significantly increased via the application of 100 µM SNP. In addition, the application of 100 µM SNP rescued agronomic traits such as plant height (24%), number of tillers per plant (40%), and visible green pigments (44%) when the plants were exposed to 1.2 mM Pb stress. Furthermore, after exposure to 1.2 mM Pb stress, the expression of the heavy-metal stress-related genes *OsPCS1* (44%), *OsPCS2* (74%), *OsMTP1* (83%), *OsMTP5* (53%), *OsMT-I-1a* (31%), and *OsMT-I-1b* (24%) was significantly enhanced via the application of 100 µM SNP. Overall, our research evaluates that exogenously applied 100 mM SNP protects rice plants from the oxidative damage brought on by 1.2 mM Pb stress by lowering oxidative stress markers, enhancing the antioxidant system and the transcript accumulation of HMs stress-related genes.

## 1. Introduction

Heavy metals (HMs), including lead (Pb), are released due to rapid industrialization, mining, economic growth, anthropogenic activities, and the excessive use of inorganic fertilizers, along with other agrochemicals [1,2]. Pollutants possessing heavy metals enter the soil via a wide range of routes and pose a threat to the sustainable agroecosystem, farming, and livelihood [3,4]. Pb remains stable in soil for a long period of time, does not easily dissociate, and hence accumulates in human and animal bodies through the consumption of contaminated plants [5], thus threatening their health. For example, it is estimated that a Pb concentration of more than 40μg dL^−1^ in an infant’s blood can cause a blockage of hemoglobin synthesis, resulting in anemia [6]. Pb is ranked the second most toxic HM after arsenic (As) [7] and has a promising role in the impairment of plant growth and development by adversely affecting the plant’s metabolism [8], seed germination, seedling growth, cell division, the permeability of plasma membrane, and various ultrastructural modifications [9,10]. A high concentration of lead (1.2 mM) induced a significant reduction in the plant height, number of tillers, number of panicles per plant, and the number of spikelets per panicle in different rice cultivars [11]. However, different rice cultivars responded differently to the 1.2 mM Pb stress. However, some species showed the lowest drop in agronomic attributes to different stresses [12].

An elevated level of Pb stimulates ROS generation, which induces oxidative stress, damages the plasma membrane, and alters metabolism and physiological reactions [13]. To cope with oxidative stress, plants have developed a sophisticated antioxidant defense mechanism comprising the generation of SOD and PO [14,15,16], APX, PPO, and CAT [17,18]. Furthermore, when plants are exposed to HMs, they use their inherent complex mechanisms and strategies for metal uptake, storage, transportation, detoxification, elimination, and compartmentalization [19,20]. However, a variation exists among different species or varieties of a species in the uptake, translocation, and accumulation of Pb [21]. In addition, phytochelatins (PCs) are the prime inducers of responses to the vulnerability of various heavy metals in plants [22]. They are considered to bind metals via thiolate coordination, which is involved in HMs homeostasis and detoxification. Recently, *OsPCS1* and *OsPCS2* genes have been characterized in rice [23]. In addition, the cation diffusion facilitator (CDF) genes family transports either metal ions out of the cytosol into extracellular spaces or diffuses into the vacuoles [24] and are also called metal tolerance proteins (MTPs). Similarly, metallothionein (MTs) play an important role in the detoxification of heavy metals [25], maintaining the balance of intracellular metallic ions in plants [26], the scavenging of ROS [27], and the regulation of developmental processes [28]. Furthermore, NO was awarded “molecule of the year” in 1992 and is crucial in modulating various physiological and biochemical activities in plants [29]. It is highly diffusible and participates in a wide range of abiotic stress tolerance mechanisms in plants [30]. ROS metabolism also involves the participation of NO [31]. It is noteworthy that ROS/NO interaction can cause cytotoxicity, or it can be protective, depending on the relative concentrations of ROS and NO [32]. The alleviative effect of NO on abiotic stresses in plants has been documented [33].

Rice is a necessity for life, and it has significantly impacted millions of people’s economics, diets, and cultures [34]. It is most often exposed to environmental hazards, including heavy metals such as lead, the main causes of which are the overuse of agrochemicals and repeated use of waste and sewage water during rice cultivation [35]. The European chemicals agency (ECHA) has assorted Pb in the group of chemicals of great perturbation for the environment [36]. Therefore, it is essential to identify the techniques that help improve the defense system of the rice against Pb stress. For example, NO applications decrease the uptake of Pb in *Arabidopsis thaliana* [37] and affect gene expression in *Zea mays* [38]. Therefore, in the current study, we investigated alleviating the toxic effects of lead on rice by applying sodium nitroprusside (SNP). The novelty of the present work is revealing the antioxidant machinery of NO in controlling Pb-induced oxidative damage in rice.

## 2. Results

### 2.1. SNP Improves Morphological Parameters of Rice under Pb Stress

**Figure 1:**
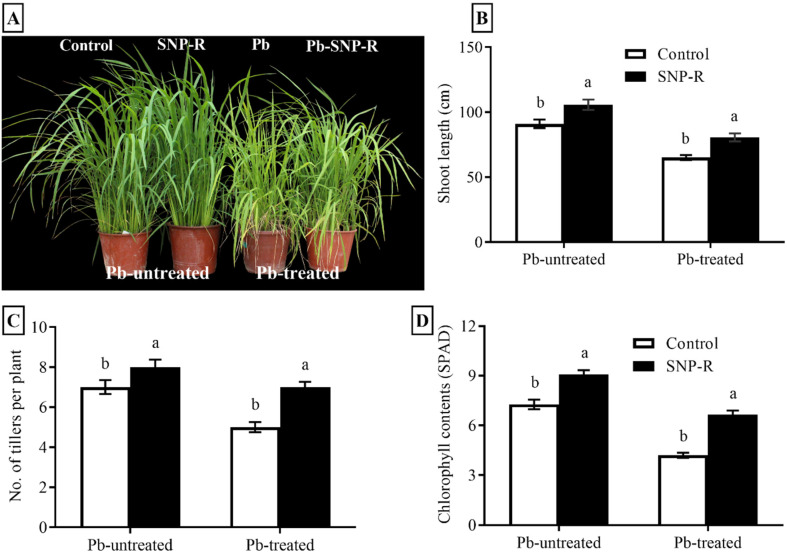
Effects of exogenously applied SNP on rice with or without Pb-induced stress. (**A**) Phenotypic characteristics; (**B**) shoot length; (**C**) number of tillers per plant; (**D**) SPAD value for photosynthetic green pigment contents. Each data point indicates the mean ± standard deviation (*n* = 3). Bars with different letters indicate significant differences, according to Duncan’s multiple range test. The results are compared to the respective controls (Pb-untreated and Pb-treated plants).

Pb-stress adversely affects the growth attributes of different crops. Our results showed a significant improvement (16%) in the plant`s shoot length under SNP treatment in Pb-untreated plants compared to Pb-untreated control plants, as shown in Figure 1A,B. The results for plants under Pb stress revealed a significant reduction in shoot length (29%) compared to the control (only water). However, SNP-treated plants under Pb stress significantly enhanced (24%) the overall shoot length compared to sole Pb-treated rice plants (Figure 1A,B). Furthermore, a considerable increase in the number of tillers (14%) in NO-treated plants was observed in Pb-untreated plants compared to their respective control plants (Figure 1A,C). Pb-treatment significantly reduced the number of tillers (29%) in Pb-treated plants compared to control Pb-untreated plants. However, SNP treatment in Pb-treated plants significantly improved the number of tillers (40%) compared to sole Pb-treated plants, as shown in Figure 1A,C. In addition, our results revealed that Pb-untreated plants supplied with SNP considerably enhanced (13%) green pigment content compared to the control Pb-untreated plants (Figure 1D). Pb stress showed a significant reduction (27%) in the visible green pigment compared to control Pb-untreated plants (Figure 1D). However, the treatment of SNP in plants under Pb stress revealed a significant increase (44%) in visible green pigment content compared to sole Pb-treated plants (Figure 1D).

### 2.2. SNP Enhances the Chlorophyll a, b, and Protein Contents

**Figure 2:**
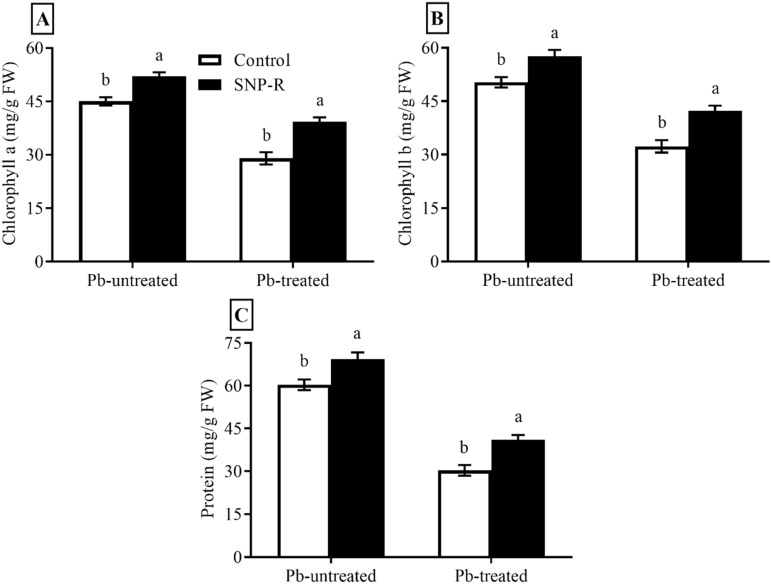
Effect of exogenously applied SNP on rice with or without Pb-induced stress. (**A**) Chlorophyll a content; (**B**) chlorophyll b content; (**C**) protein content. Each data point indicates the mean ± standard deviation (*n* = 3). Bars with different letters indicate significant differences, according to Duncan’s multiple range test. The results are compared to the respective controls (Pb-untreated and Pb-treated plants).

Plants under stress face the challenge of inhibition in photosynthesis and changes in chlorophyll contents. The results revealed that SNP applications considerably increased chlorophyll a and b levels (16 and 15%, respectively) in Pb-untreated plants compared to control Pb-untreated plants, as shown in Figure 2A,B. Plants under Pb stress showed a significant decrease (36 and 34%, respectively) in chlorophyll a and b contents compared to control Pb-untreated plants (Figure 2A,B). The data also showed a significant increase in chlorophyll a and b contents (38 and 36%, respectively) in SNP-treated plants under Pb stress compared to plants under sole Pb stress (Figure 2A,B). Furthermore, the obtained data showed a significant increase in protein content (15%) in SNP-supplied Pb-untreated plants compared to control Pb-untreated plants, as shown in Figure 2C. Plants under Pb-treatment showed a highly significant decrease (49%) in protein content compared to control Pb-untreated plants (Figure 2C). However, SNP applications in Pb-treated plants significantly increased the total protein content (35%) in Pb-treated plants compared to sole Pb-treated plants (Figure 2C).

### 2.3. Exogenously Applied SNP Mitigates Membrane Injury and Enhances Protection of Rice against Pb-Toxicity

**Figure 3:**
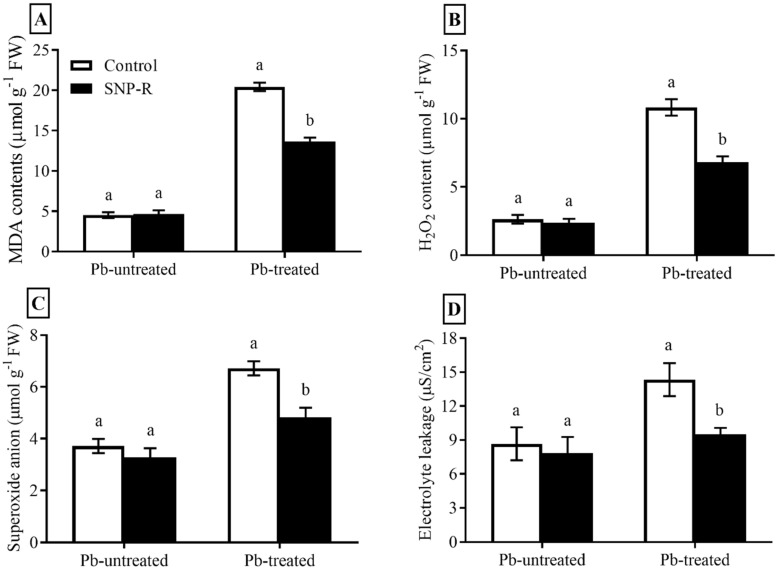
Effect of exogenously applied SNP on rice with or without Pb-induced stress. (**A**) MDA level; (**B**) H_2_O_2_ content; (**C**) superoxide anion level; (**D**) electrolyte leakage. Each data point indicates the mean ± standard deviation (*n* = 3). Bars with different letters indicate significant differences, according to Duncan’s multiple range test. The results are compared to the respective controls (Pb-untreated and Pb-treated plants).

In the current study, we compute the impact of SNP on ROS compounds, including H_2_O_2_, O_2_^−^, MDA, and electrolyte leakage. Pb-treatment significantly enhanced (305%) MDA levels in plants compared to control Pb-untreated plants (Figure 3A). However, the SNP supply in Pb-treated plants significantly reduced (33%) MDA levels in Pb-treated plants compared to sole Pb-treated plants (Figure 3A). A significant increase in ROS (H_2_O_2_ and O_2_^−^) levels (311 and 81%, respectively) was observed in Pb-treated plants compared to control Pb-untreated plants (Figure 3B,C). However, SNP applications considerably mitigate H_2_O_2_ and O_2_^−^ levels (37 and 28%, respectively) in plants under Pb stress compared to plants under sole Pb stress (Figure 3B,C). Furthermore, Pb treatments significantly enhanced (65%) ion leakage compared to control Pb-untreated plants (Figure 3D), which was significantly mitigated (34%) by SNP applications in Pb-treated plants, as shown in Figure 3D.

### 2.4. SNP Regulates the Antioxidant Enzymes Machinery

**Figure 4:**
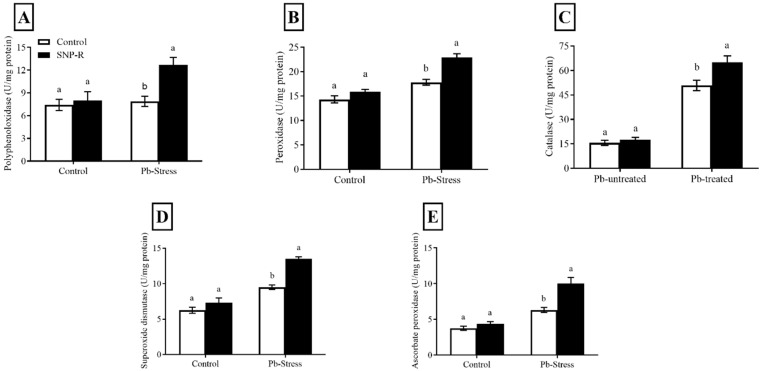
Effect of exogenously applied SNP on rice with or without Pb-induced stress. (**A**) Polyphenol oxidase; (**B**) peroxidase; (**C**) catalase; (**D**) superoxide dismutase; (**E**) ascorbate peroxidase. Each data point indicates the mean ± standard deviation (*n* = 3). Bars with different letters indicate significant differences, according to Duncan’s multiple range test. The results are compared to the respective controls (Pb-untreated and Pb-treated plants).

Overall, the results revealed that NO treatments increased the level and activity of antioxidant enzymes under Pb stress. The activities of PPO, PO, CAT, SOD, and APX were enhanced (35, 18, 15, 17, and 44%, respectively) in SNP-supplemented Pb-untreated plants compared to control Pb-untreated plants, as shown in Figure 4A–E. Pb treatment considerably activates the production of PPO, PO, CAT, SOD, and APX (7, 24, 218, 52, and 68%, respectively) compared to control Pb-untreated plants (Figure 4A–E). However, the supply of SNP significantly enhanced the activity of the above antioxidants by 60, 28, 26, 42, and 58%, respectively, compared to sole Pb-supplied plants.

### 2.5. SNP Modulates the Expression of the Genes Related to Metal Stress

**Figure 5:**
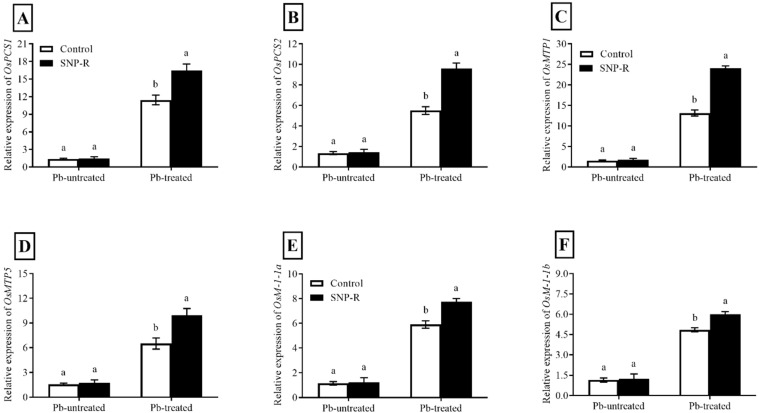
Effect of exogenously applied SNP on the relative expression of rice phytochelatin, metal transporter, and metallothionein protein candidate genes with or without Pb-induced stress. (**A**) *OsPC1*; (**B**) *OsPC2*; (**C**) *OsMTP1*; (**D**) *OsMTP5*; (**E**) *OsM1-1a*; (**F**) *OsM1-1b*. Each data point indicates the mean ± standard deviation (*n* = 3). Bars with different letters indicate significant differences, according to Duncan’s multiple range test. The results are compared to the respective controls (Pb-untreated and Pb-treated plants).

The results revealed that the relative expression of *OsPCS1* and *OsPCS2* is differentially and highly significantly enhanced (387 and 134%, respectively) in Pb-treated plants compared to the Pb-untreated control. However, SNP supplementation in Pb-treated plants shows significant improvements in the relative expression of *OsPCS1* (44%) and *OsPCS2* (74%) compared to sole Pb-supplied plants (Figure 5A,B).

Furthermore, our results showed that plants under Pb stress have significantly higher relative *OsMTP1* and *OsMTP5* expression (460 and 177%, respectively) when compared to control Pb-untreated plants. However, the SNP supply further improved the relative expression of *OsMTP1* and *OsMTP5* (83 and 53%, respectively) compared to sole Pb-treated plants (Figure 5C,D).

Pb treatments triggered a significant enhancement in relative expressions of *OsMT-I-1a* and *OsMT-I-1b* (151 and 1065%, respectively) compared to Pb-untreated control plants (Figure 5E,F)*. However,* SNP supplies further improved the relative expression of *OsMT-I-1a* and *OsMT-I-1b* (31 and 24%, respectively) compared to plants solely treated with Pb.

## 3. Discussion

Nitric oxide acts as a “guardee” molecule to alleviate heavy-metal toxicities via stress perception, signaling, and the acclimatization of plants under heavy-metal stress [39]. In the current study, the observed inhibitory effects of Pb on key morphological characteristics of rice, i.e., plant height, the number of tillers, and visible green pigment content (Figure 1A–D), were significantly reduced by an exogenous application of SNP in the form of 100 µM SNP solutions. Similarly, the toxic effects of Pb on wheat seedling growth were significantly reduced via the application of 100 µM SNP [40].

Many findings reported changes in the physiological, biochemical, and molecular levels of plants, such as the accumulation and activity of chlorophyll a and b contents in plants under abiotic stress [41,42,43,44,45,46]. SNP significantly ameliorated Pb-induced negative impacts on chlorophyll a and b contents, as shown in Figure 2A,B. Similarly, under heavy-metal (Pb and Cd) stress, adding SNP increases chlorophyll and carotenoid concentrations in bamboo plants [47].

Plants activate a plethora of adaptive responses to withstand abiotic stresses, including a high accumulation of proteins [48]. The current study showed that the significant reduction in protein content due to Pb-induced damages is significantly mitigated by exogenous application of SNP, as shown in Figure 2C. This result is in accordance with the previous reported result [47].

Heavy metals cause damage at the cellular and molecular levels to plants, both directly and indirectly, through overproduction and the hyperaccumulation of ROS [49,50]. However, NO protects plants against oxidative damage by scavenging ROS [49]. In the present study, due to Pb stress, a significant enhancement in the production of ROS and electrolyte leakage was observed. However, it was significantly mitigated by exogenously applied SNPs (Figure 3A–D). These results are in accordance with the previous literature published [51,52]. Exogenous applications of NO in rice and perennial ryegrass under Cd and Pb-induced stress, respectively, decreased the production of ROS and MDA, resulting in increased activities of antioxidant enzymes [53,54,55].

NO regulates the antioxidant enzyme machinery at the cellular level, affecting the cellular redox level [49,56]. Our results indicated that the application of NO exogenously in the form of SNP mitigates the adverse effects of Pb on rice plants by increasing the production of antioxidant enzymes such as PPO, POD, CAT, SOD (Figure 4A–D), and APX (Figure 4E), which is according to previous literature published for various heavy metals [57].

The increased tolerance to heavy metals is linked with phytochelatin synthesis, for example, cadmium stress [58,59]. Several published reports suggest that PC-deficient mutants increased heavy-metal sensitivity [60]. Therefore, the current study was designed to check the effect of the exogenous application of SNP on the expression of two candidate phytochelatins, i.e., *OsPCS1* and *OsPCS2*. Our results showed that the relative expression of both genes was significantly enhanced under lead stress compared to control plants (Figure 5A,B), suggesting the transcript’s accumulation for metal binding and vacuolar compartmentalization. To elucidate if NO affects the expression of phytochelatin genes, the observed effects and data showed that the expression levels of transcripts were considerably enhanced in SNP-supplied Pb-treated plants compared to plants under sole Pb stress.

Plants under heavy-metal stress undergo a transcriptional regulation of CDF protein family members *OsMTP1* and *OsMTP5*. They play an important role in cation homeostasis, chelation, sequestration, or the expulsion of excess heavy metals [61]. The expression pattern of *OsMTP1* is enhanced during Cd-stress exposure; overexpression and gene silencing confirmed its role in the transportation of Cd [62]. The expression analyses of *OsMTP1 and OsMTP5* in the current study for Pb stress are tallied with the results in the cited literature. The current study revealed improved expressions of *OsMTP1 and* *OsMTP5* in plants treated with Pb compared to control Pb-untreated plants, as shown in Figure 5C,D. To elucidate if SNP affects the relative expression of *OsMTP1 and* *OsMTP5*, the results showed that the expression level was significantly enhanced in NO-treated rice under Pb stress compared to rice only under Pb stress (Figure 5C,D).

The overexpression of metallothionein genes in tobacco showed decreased and increased Arsenic accumulation in roots and shoots, respectively [63]. In the current study, we found that the relative expression of two candidate genes, i.e., *OsMT-I-1a* and *OsMT-I-1b*, is upregulated by Pb toxicity, as shown in Figure 5E,F, which is further improved by the application of SNP in Pb-treated plants. Although the mechanism of heavy-metal detoxification in plants by metallothionein is still elusive, our results are supported by the previous published literature [64].

## 4. Materials and Methods

### 4.1. Plant Material, Husbandry Preparation, and Growth Conditions

The experiment was performed in soil under greenhouse conditions at Kyungpook National University, Daegu, Republic of Korea. For the experiment, the Jinbu rice cultivar *(Oryza sativa* L. ssp. *Japonica)* was selected as genetic material. Seeds sterilization, germination, and sowing were conducted as previously described [65]. Lead (II) nitrate (Pb(NO_3_)_2_) measuring 1.2 mM was applied as described earlier [11]. The pots were divided into four treatments with three replicates each, as listed in Table 1. After three weeks of transplantation, the plants were supplied with 100 µM SNP [40,54].

### 4.2. Measurement of Electrolyte Leakage and Visible Green Pigment Quantification

The electrolyte leakage assay was performed to estimate any ion leakage that would have resulted from Pb oxidative damage, as previously described [48]. Electrolyte leakage-1 (EL1) was measured using a portable conductivity meter (HURIBA Twin Cond B-173, Fukuoka, Japan). For electrolyte leakage-2 (EL2), the samples were autoclaved and cooled at room temperature. Electrolyte leakage (EL), expressed in percentage (%), was calculated using the following formula.

EL% = EL1/EL2 × 100

Visible green pigment content in leaves was measured in leaf samples using a SPAD meter (SPAD-502; Minolta Co. Ltd., Osaka, Japan), as previously described [11].

### 4.3. Quantification of Chlorophyll a and b Contents

To quantify and calculate chlorophyll a and b contents, a detailed method was followed as described previously [66,67].

### 4.4. Quantification of Lipid Peroxidation

The determination of lipid peroxidation was performed by calculating the amount of a byproduct of membrane bilayer oxidation, malondialdehyde (MDA), by using a published method [68].

### 4.5. Hydrogen Peroxide (H_2_O_2_) and Superoxide Anion (O_2_^−^) Content

H_2_O_2_ content in rice leaf tissue was quantified and calculated using the method described [69] and expressed in units as μmol g^−1^ FW. Similarly, O_2_^−^ contents in rice leaf tissue were quantified and calculated as previously described [70] and expressed in units as μmol g^–1^ FW.

### 4.6. Estimation of Antioxidant Activities

As previously mentioned, CAT, POD, PPO, and SOD activity were examined [48]. In brief, 400 mg of leaf samples was powdered using a chilled mortar and pestle. The crushed samples were homogenized with 0.1 M phosphate buffer (pH 6.8) and centrifuged at 4 °C for 15 min at 5000 rpm. The supernatant was used as the crude enzyme source for CAT, POD, and PPO activities.

The activity of PPO and POD was estimated [48], and the activity of CAT was measured [11] as previously described. Furthermore, SOD activities were analyzed by following the photoreduction of nitro blue tetrazolium (NBT) [66], and the APX’s activity was determined [71], as mentioned earlier.

### 4.7. RNA Extraction and Quantitative Real-Time PCR

RNA was extracted using the standardized procedure followed by [67]. Additionally, complementary DNA (cDNA) and RT-PCR were carried out according to the previous literature [72]. The synthesized cDNA was used as a template for further assessments of transcript accumulation using qRT-PCR (Eco™ Illumina™ San Diego, California, USA), as previously described [29]. The list of genes and corresponding primers with names and sequences is provided in Table 2.

### 4.8. Statistical Analysis

Independent experimental analyses were performed in triplicates using a completely randomized design (CRD). To assess the statistical significance between Pb-treated plants with control and SNP-supplied Pb-plants, Statistical Analysis Software SAS version 9.1 (SAS Institute Inc., Cary, NC, USA) was used, and all data were statistically evaluated with Duncan’s multiple range test. The significance threshold was set at *p* < 0.05. GraphPad Prism software version 6.0 (San Diego, CA, USA) was used to present the results graphically.

## 5. Conclusions

By altering the activities of SOD, POD, and PPO, as well as the amount of CAT and gene expression, Pb stress induced the overproduction of ROS and disrupted the H_2_O_2_ and MDA scavenging system. Conclusively, the significant role of exogenous NO donors (SNP) in plants has a protective effect in alleviating lead stress in rice. Our results revealed that exogenously applied SNP improves the Pb stress tolerance in rice by activating the antioxidant system, lowering electrolyte leakage, reducing the production of H_2_O_2_ and MDA, and increasing the expression of heavy-metal stress-related genes. Based on the present results, it is suggested that the application of SNP will enhance the growth and productivity of rice under lead stress conditions.

## Figures and Tables

**Table 1:** Demonstrates different treatments and concentrations of the chemicals used in this study.

Treatments	Concentrations
Control	untreated
SNP-R	100 µM SNP
Pb	1.2 mM Pb (NO_3_)_2_
Pb + SNP-R	1.2 mM Pb (NO_3_)_2_ + 100 µM SNP

Pb(NO_3_)_2_: Lead (II) nitrate as a Pb source; SNP-R: sodium nitroprusside as a NO-donor applied through roots via sub-irrigation.

**Table 2:** List of primers used in this study.

Primer	Forward Sequence (5′-3′)	Reverse Sequence (5′-3′)
*OsActin*	GGA ACT GGT ATG GTC AAG GC	AGT CTC ATG GAT AAC CGC AG
*OsPCS1*	CGA AGA TTC CAT TTC CCA GA	TCG AGG ATA TCG GTG AAA GC
*OsPCS2*	TCC CTC TCC GTC GTC CTC	CCT CCG CCT TCA CCT TGT
*OsMTP1*	TCA AGA TGC TGC GCA ACA TCC	GAG CTC CTA CTC GCG CTC AAT G
*OsMTP5*	ACG CTC GTT GTC TGA TGG G	GTC ACT GCA AGC ATG ATG TCC AC
*OsMT-I-1a*	TGC GGA AG TAC CCT GA	TTC TCC GGC GCC ACA C
*OsMT-I-1b*	CTG TGG ATC AAG CTG TGG CT	GCT GCT GCT CTT CTC TTC CA

## Data Availability

Not applicable.
